# Loss of microRNA-30a and sex-specific effects on the neonatal hyperoxic lung injury

**DOI:** 10.1186/s13293-023-00535-6

**Published:** 2023-08-08

**Authors:** Sandra L. Grimm, Samuel Reddick, Xiaoyu Dong, Connor Leek, Amy Xiao Wang, Manuel Cantu Gutierrez, Sean M. Hartig, Bhagavatula Moorthy, Cristian Coarfa, Krithika Lingappan

**Affiliations:** 1 Dan L Duncan Comprehensive Cancer Center, Baylor College of Medicine, Houston, TX USA; 2 Center for Precision Environmental Health, Baylor College of Medicine, Houston, TX USA; 3 Molecular and Cellular Biology Department, Baylor College of Medicine, Houston, TX USA; 4 Vanderbilt University School of Medicine, Nashville, TN USA; 5 Department of Pediatrics, Baylor College of Medicine, Houston, TX USA; 6 Department of Pediatrics, Division of Neonatology, Children’s Hospital of Philadelphia, University of Pennsylvania, Philadelphia, PA USA; 7 Department of Medicine, Division of Endocrinology, Baylor College of Medicine, Houston, TX USA

**Keywords:** Bronchopulmonary dysplasia, Micro-RNA, Newborn, Lung, Hyperoxia, Oxidative stress

## Abstract

**Background:**

Bronchopulmonary dysplasia (BPD) is characterized by an arrest in lung development and is a leading cause of morbidity in premature neonates. It has been well documented that BPD disproportionally affects males compared to females, but the molecular mechanisms behind this sex-dependent bias remain unclear. Female mice show greater preservation of alveolarization and angiogenesis when exposed to hyperoxia, accompanied by increased *miR-30a* expression. In this investigation, we tested the hypothesis that loss of *miR-30a* would result in male and female mice experiencing similar impairments in alveolarization and angiogenesis under hyperoxic conditions.

**Methods:**

Wild-type and *miR-30a*^−/−^ neonatal mice were exposed to hyperoxia [95% FiO_2_, postnatal day [PND1-5] or room air before being euthanized on PND21. Alveolarization, pulmonary microvascular development, differences in lung transcriptome, and *miR-30a* expression were assessed in lungs from WT and *miR-30a*^−/−^ mice of either sex. Blood transcriptomic signatures from preterm newborns (with and without BPD) were correlated with WT and *miR-30a*^−/−^ male and female lung transcriptome data.

**Results:**

Significantly, the sex-specific differences observed in WT mice were abrogated in the *miR-30a*^−/−^ mice upon exposure to hyperoxia. The loss of *miR-30a* expression eliminated the protective effect in females, suggesting that *miR-30a* plays an essential role in regulating alveolarization and angiogenesis. Transcriptome analysis by whole lung RNA-Seq revealed a significant response in the *miR-30a*^−/−^ female hyperoxia-exposed lung, with enrichment of pathways related to cell cycle and neuroactive ligand–receptor interaction. Gene expression signature in the *miR-30a*^*−/−*^ female lung associated with human BPD blood transcriptomes. Finally, we showed the spatial localization of *miR-30a* transcripts in the bronchiolar epithelium.

**Conclusions:**

*miR-30a* could be one of the biological factors mediating the resilience of the female preterm lung to neonatal hyperoxic lung injury. A better understanding of the effects of *miR-30a* on pulmonary angiogenesis and alveolarization may lead to novel therapeutics for treating BPD.

**Supplementary Information:**

The online version contains supplementary material available at 10.1186/s13293-023-00535-6.

## Plain Language Summary

Bronchopulmonary dysplasia (BPD) is a lung condition that affects babies born prematurely, causing problems with their lung development. Interestingly, BPD tends to affect boys more than girls, but we do not fully understand why. To investigate this, we conducted a study using mice. Female mice had better lung development and blood vessel formation when exposed to high oxygen levels. We found higher expression of a molecule called *miR-30a* in the female mice and seemed to be protective. So, we wanted to see if removing *miR-30a* would have the same effect on both male and female mice. To test this, we exposed newborn mice without *miR-30a* and normal mice to high oxygen levels or regular room air. Interestingly, the differences between normal males and females were no longer present in the mice without *miR-30a*. This suggested that *miR-30a* plays an important role in lung development. We also identified that the female mice without *miR-30a,* when exposed to high oxygen, had the greatest number of genes affected, and these gene changes were like those seen in blood samples from premature babies with BPD. Finally, we report that *miR-30a* was in a specific part of the lung called the bronchiolar epithelium. Overall, this study suggests that *miR-30a* is crucial in protecting premature lungs from damage caused by high oxygen levels. By understanding how *miR-30a* affects lung development, we may be able to develop new treatments for BPD in the future.

**Supplementary Information:**

The online version contains supplementary material available at 10.1186/s13293-023-00535-6.

## Highlights

- Sex-specific differences are abrogated in neonatal *miR-30a*^−/−^ mice upon exposure to hyperoxia.
- The loss of *miR-30a* expression eliminated the female resilience to neonatal hyperoxic lung injury.
- Pulmonary *miR-30a* expression could be one of the protective epigenetic mechanisms in the neonatal female lung.

**Supplementary Information:**

The online version contains supplementary material available at 10.1186/s13293-023-00535-6.

## Background

Bronchopulmonary dysplasia (BPD) is characterized by an arrest in lung development with severe impairment in vascular development and alveolarization [1]. Despite recent advances in perinatal care, BPD remains a leading cause of morbidity in premature neonates [2, 3]. Infants with BPD also face a variety of long-term sequelae, including pulmonary hypertension, increased rehospitalization rates, and additional risk for pulmonary disease as adults [4–8]. Many pre- and post-natal factors contribute to disease pathogenesis in BPD, including exposure to postnatal hyperoxia due to the generation of reactive oxygen species [9, 10]. It has been well documented that BPD disproportionally affects males compared to females. Males are at an increased risk for developing moderate-to-severe BPD and experience higher neonatal and infant mortality rates, but the molecular mechanism behind this sex-dependent bias remains unclear [11–18].

MicroRNAs (miRs) play an important role in the post-transcriptional regulation of protein-coding genes and act primarily by destabilizing the mRNA of their target genes. *miR-30a* is a known pro-angiogenic miR that stimulates arteriolar branching via the downregulation of *Dll4* [19, 20]. *miR-30a* levels are decreased in the peripheral blood of neonates with evolving and established BPD, which may be correlated with arrested lung development [21].

**Fig. 1:**
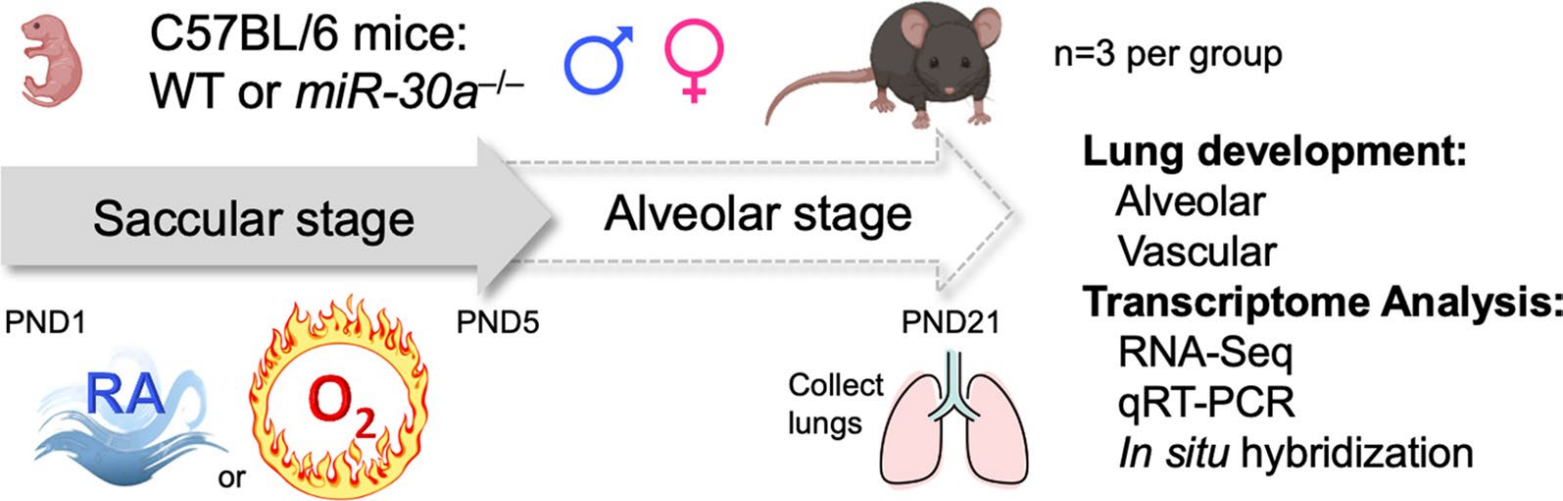
Loss of miR-30a causes loss of resilience in the female neonatal mice in a model of neonatal hyperoxic lung injury. Schematic showing the exposure of WT and *miR-30a*^−/−^ neonatal mice to hyperoxia (95% FiO_2_, PND1-5) during the saccular stage of lung development. Euthanasia was performed at PND21 with evaluation of alveolarization, pulmonary vascular development, and assessment of the pulmonary transcriptome

Mice exposed to postnatal hyperoxia develop pathognomonic features of human BPD and are used to model the disease [22–24]. Like their human counterparts, female mice are more resilient when exposed to hyperoxia, while males show greater arrest in alveolarization and angiogenesis [18, 25]. Previous studies found that this sexual dimorphism is accompanied by an increased pulmonary expression of microRNA-30a (*miR-30a*) in female mice compared to male mice exposed to hyperoxia. The findings were replicated in neonatal human pulmonary microvascular endothelial cells (HPMECs), with female endothelial cells showing higher expression of *miR-30a* when exposed to hyperoxia in vitro [26]. *miR-30a* could contribute to the resilience of the neonatal female lung when exposed to hyperoxia. In this investigation, we sought to clarify the role *miR-30a* plays in the previously observed sex-specific differences in lung development of mice exposed to hyperoxia*.* We tested the hypothesis that loss of *miR-30a* in mice would result in male and female mice experiencing similar impairments in alveolarization and angiogenesis when exposed to hyperoxia. The current study outlines the consequences of loss of *miR-30a* in neonatal hyperoxic lung injury, the sex-specific differences, and reveals the biological pathways mediating these differences, as outlined in Fig. 1. Overall, our results reveal a critical role of *miR-30a* in possibly mediating the sex-specific differences in BPD.

## Materials and methods

### Animals

All animal studies were conducted with the approval of the Institutional Animal Care and Use Committee (IACUC) of Baylor College of Medicine. Experiments were performed in accordance with relevant guidelines and regulations. Care of animals in research met the highest contemporary standards per the 8^th^ edition of the *Guide for the Care of Use of Laboratory Animals* and other IACUC protocols. *miR-30a*^−/−^ mice were received from Dr. Sean Hartig from Baylor College of Medicine. CRISPR/Cas9 gene editing was used to knockout the entire *miR-30a* gene. Wild-type (WT) and *miR-30a*^*−/−*^ mice were bred in the animal facility and both were on the C57BL/6J background. Guide RNAs flanked the 1-kb genomic region that contains *miR-30a* and knockout was confirmed using qPCR analysis and DNA sequencing of the deleted region. The *miR-30a*^*−/−*^ mice are viable and show no overt phenotypes during postnatal growth [27]. The sex of the neonatal mouse pups was determined by both anogenital distance and pigmentation in the anogenital region [28]. Sex was reconfirmed with PCR analysis for the *Sry* gene in genomic DNA obtained from mouse tail clips [18].

### Mouse model of BPD

Exposure to hyperoxia (95% FiO_2_) mimics the alveolar arrest of bronchopulmonary dysplasia in mouse pups [24, 29]. Mouse pups from multiple litters were pooled before being randomly and equally redistributed by sex into two groups, one group exposed to normoxia (21% FiO_2_), and the other group exposed to hyperoxia (95% FiO_2_), within 12 h of birth for 5 days [23]. Animals were chosen at this time because neonatal mice are at the saccular stage of lung development during this period, which is approximately equivalent to 26–36 weeks in human neonates. The dams were rotated between air- and hyperoxia-exposed litters every 24 h to prevent oxygen toxicity and to eliminate maternal effects between the groups. Oxygen exposure was conducted in plexiglass chambers as previously described [18]. Mice were euthanized on postnatal day (PND) 21 after recovery in room air, as most of postnatal lung development in mice is complete by this age. *miR-30a* levels were most different between male and female mice after early hyperoxia exposure at PND21 with female mice showing significantly higher expressions. Hence, PND21 was chosen as the time-point for analysis of lung morphometry and gene expression signatures [26]. The control group was kept at room air for the same duration of time. 5–7 pups/genotype/treatment were included for the morphometry related studies. Data from previously published studies were used in this manuscript, including lung histology data in WT mice [18] and lung transcriptome data from male and female WT neonatal mice [25]. The WT and *miR-30a*^*−/−*^ mice were not exposed to hyperoxia simultaneously for this study.

### Lung histology

Both hyperoxia- and normoxia-exposed mice were anesthetized (100 mg/kg i.p. pentobarbital sodium), tracheas were cannulated, and lungs were fixed by instilling with 4% paraformaldehyde endotracheally at 25 cmH_2_O pressure for 15 min. The trachea was tied off and the lungs were removed and further fixed overnight at 4 °C, followed by dehydration in graded alcohol and embedding in paraffin.

### Analysis for alveolarization

Sections (5 μm) were prepared and stained with hematoxylin and eosin (H&E). Alveolar development was evaluated by the measurement of mean linear intercept (MLI) [30]. Ten randomly selected, non-overlapping areas were photographed with bright field microscopy (40×). The photographs were overlaid with a grid and alveolar wall intersections with array lines were manually counted in a blinded fashion to prevent observer bias. Fields containing large airways and vessels were excluded. Radial alveolar count was measured as previously described [18]. Each section was analyzed in a blinded fashion to prevent observer bias by two reviewers.

### Analysis for macrophages

Pulmonary macrophage count was determined based on immunofluorescence staining for F4/80 (1:500 dilution, Bio-Rad Laboratories; catalog no. MCA497GA), a macrophage-specific marker. Fifteen randomly selected, non-overlapping areas were photographed with bright field microscopy (40×). For each image captured, brown-color-stained macrophages were manually identified and counted to determine the macrophage count [18]. Each section was analyzed in a blinded fashion to prevent observer bias by two reviewers.

### Analysis for pulmonary vascular development

Pulmonary vessel density was determined based on immunofluorescence staining for von Willebrand factor (vWF; 1:4000 dilution, Abcam, ab6994), an endothelial specific marker. Ten randomly selected, non-overlapping areas were photographed with bright field microscopy (20 ×). For each image captured, pulmonary vessels (< 50 um in diameter) were manually identified and counted to determine density [18]. Each section was analyzed in a blinded fashion to prevent observer bias by two reviewers.

### Preparation of lung samples for transcriptomic analysis, library preparation, and sequencing

Mice were euthanized on PND21 with i.p. pentobarbital. The right ventricle was perfused with ice cold PBS, and lungs were flash frozen in liquid nitrogen until ready for total RNA extraction. Total RNA from frozen lung samples was isolated using the Zymo micro kit (Zymo Research, Irvine, CA). Sample concentration was assayed using Nanodrop-8000 (Thermo Scientific, Wilmington, DE), and quality checks were done using the NanoDrop spectrophotometer and the Agilent Bioanalyzer. Three samples of total RNA with RIN values > 8, were subjected to RNA-Seq analysis. RNA libraries were prepared with QIAseq Stranded Total RNA library Kit (Qiagen) using 1000 ng input RNA, and purity of the libraries was analyzed using the DNA 1000 tape Tapestation 4200 (Agilent). The indexed libraries were pooled and sequenced.

### Analysis of RNA-Seq data

The *miR-30a*^*−/−*^ data have been submitted to NCBI GEO (GSE213287). Previous WT samples were submitted to NCBI GEO (GSE97804). All the RNA-Seq used was trimmed for Illumina adapters and low-quality terminal base pairs using trim galore. Filtered and trimmed sequences were mapped to the mouse genome build UCSC mm10 using the STAR software [31]. Gene expression was quantified as read counts using featureCounts [32]. Further batch normalization was applied using CombatSeq [33]. Data were normalized using RUVr [34] and upper quartile normalization; the EdgeR [35] R package was used for differential gene expression analysis, with significance achieved at FDR < 0.05 and fold change exceeding 1.5x. Enriched pathways were determined using Gene Set Enrichment Analysis (GSEA) against gene set collections compiled by MSigDB [36], with significance achieved at FDR < 0.25 per the software developers' best practices. Heatmaps were generated using the R statistical system. Upset plots for gene signatures were generated using the ComplexHeatmap R package.

### Analysis using GTex data

Transcriptomic profiles of 578 whole adult human lungs compiled by the Genotype-Tissue Expression (GTEx) project were downloaded. Gene symbols conserved between mouse and human were converted to the mouse symbols using Biomart. Gene signatures for each sample were computed using the summed z-scores method. First expression of each gene across all samples was converted to a z-score to determine how many standard deviations each gene was away from the mean. Next, for each sample z-scores of up-regulated genes were added, and z-scores of down-regulated genes were subtracted. Gene signature correlation was computed for all signatures using the Pearson correlation coefficient as implemented in the Python scientific library, with significance achieved for p-value < 0.05 [37].

### Analysis using the human blood data

Bronchopulmonary dysplasia (BPD) blood transcriptomics from newborns were downloaded from the dataset GSE32472 [38]. Gene symbols for genes conserved between mouse and human were converted to the mouse gene symbols using Biomart. Gene signatures for each sample were computed using the summed z-scores method as described above. Correlations of summed z-scores with numerical clinical variables were computed using the Python scientific library.

### Quantitative real-time PCR (qRT-PCR)

Total RNA was extracted from the lungs using the Quick-RNA MiniPrep kit (ZYMO Research, Cat# R1055) following manufacturer's instruction. RNA integrity was evaluated by Agilent 2100 bioanalyzer. cDNA was synthesized with 1 ug of total RNA using Applied Biosystems High-Capacity cDNA Reverse Transcription Kit (ThermoFisher, Cat# 4,374,966). qRT-PCR was conducted in duplicates on a 384-well plate on the Applied Biosystems QuantStudio 6 Pro system (ThermoFisher) using the Applied Biosystems TaqMan Fast Advanced Master Mix (2X) with following conditions (QuantStudio 6 Pro Fast run mode): hold 50 °C for 2 min, hold 95 °C for 20 s followed by 95 °C for 1 s and 60 °C for 20 s for 40 cycles. All gene expression assays were conducted using Applied Biosystems Taqman Probes (TaqMan® Gene Expression Assays, ThermoFisher Scientific). Genes of interest used in this study were *Chl1* (Mm00483313_m1) and *Cd109* (Mm00462151_m1). Three housekeeping genes, including *Vcl* (Mm00447745_m1), *B2m* (Mm00437762_m1) and *Tbp* (Mm01277042_m1), were selected as endogenous controls for normalization. Gene expression Ct values for both target and housekeeping genes were directly acquired by QuantStudio Design and Analysis software (DA2 software v2.6.0, ThermoFisher Scientific). The target gene expression (ΔCt value) was normalized through incorporation of the housekeeping genes *B2m/Vcl/Tbp* by the DA2 software. The changes in expression for genes of interest (ΔΔCt values) were calculated using the gene expression in room air values in the respective sex as reference, and the fold changes were generated using the 2^−ΔΔCt^ method.

### In situ hybridization

Mice were euthanized on PND21 with i.p. pentobarbital, lungs were inflated and fixed in 4% paraformaldehyde (PFA) for 24 h. Histomorphometry was carried out on 7-μm paraffin sections of samples (*n = *3 per sex/oxygen environment for WT). Samples were stained using hematoxylin and eosin (H&E), cover slipped with Vectamount permanent mounting medium (Vector labs, Newark, CA, USA), and imaged with Leica Thunder Imager DMi8 (Leica, Nußloch, Baden-Wurttemberg, Germany). On serial sections to the sections used for H&E, we performed in situ hybridization (ISH) using the BaseScope Duplex Kit (Advanced Cell Diagnostics, Hayward, CA, USA). Samples (*n = *3 per sex/oxygen environment) were treated with a *miR-30a* probe (Advanced Cell Diagnostics, Hayward, CA, USA) for 2 h at 4 °C. A positive control slide used a mixture of probes targeting housekeeping gene *Polr2A*. A negative control slide used dapB (a bacterial gene). Nuclei were counterstained with a 50% hematoxylin solution and mounted with Vectamount permanent mounting medium (Vetor labs, Newark, CA, USA). Slides were imaged using Leica Thunder Imager DMi8 (Leica, Nußloch, Baden-Wurttemberg, Germany).

### Statistical analysis

GraphPad version 9 (GraphPad Software, San Diego, CA) was used for data analysis. Data were expressed as means ± SE. Differences were assessed by three-way ANOVA to test for the independent effects of sex, genotype and hyperoxia. Multiple comparison correction (Bonferroni) was performed. Differences determined by ANOVA were considered significant if p-values were less than 0.05 after correction.

## Results

### Sex-specific differences in alveolarization were lost in miR-30a^−/−^ mice in the setting of neonatal hyperoxia exposure

**Fig. 2:**
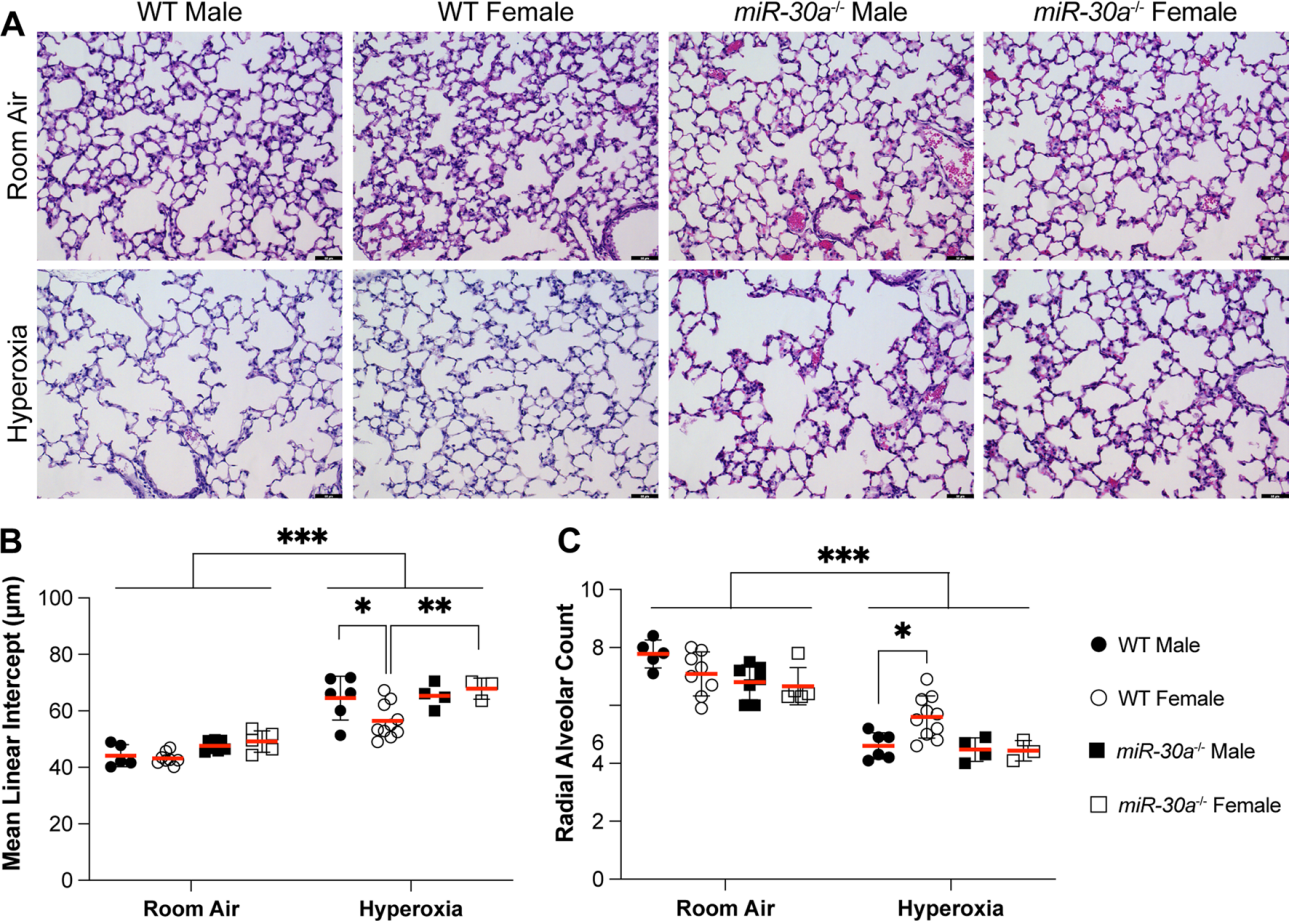
Sex-specific differences in alveolarization were lost in miR30a^−/−^ mice in the setting of neonatal hyperoxia exposure: Lung morphometry (mean linear intercept; MLI) and radial alveolar count (RAC) were measured in WT and *miR-30a*^−/−^ mice. **A** Representative hematoxylin and eosin-stained sections from male and female WT or *miR-30a*^−/−^ mice in the room air and hyperoxia groups at 40 × magnification. Black bars = 50 µm. **B** Quantitation of Mean linear intercept (MLI) or **C:** radial alveolar count (RAC) in neonatal WT and *miR-30a*^−/−^ mice exposed to room air or hyperoxia (95% FiO_2_, PND 1–5) on PND21. Values are mean ± SE. Independent biological replicates in each group are shown (*n = *3–10/group). Significant differences between groups are shown by *P < 0.05, **P < 0.01, and ***P < 0.001. Statistical analysis was performed using 3-way ANOVA

Male and female *miR-30a*^−/−^ mice were exposed to room air or hyperoxia (95% FiO_2_) within 12 h of birth for 5 days, during the saccular stage of lung development. After recovering in room air until PND21, lung morphometry was assessed to evaluate the effect of hyperoxia in the absence of *miR-30a* expression. Representative lung sections (PND21) stained with hematoxylin and eosin from WT and *miR-30a*^−/−^ mice are shown in Fig. 2A**.** Alveolar development was quantified using mean linear intercept (MLI) and radial alveolar count (RAC) shown in Fig. 2. There was no difference between WT and *miR-30a*^−/−^ mice under normoxic conditions. Statistical analysis was performed by 3-way ANOVA to account for sex, genotype, and treatment. The statistical significance of the independent variables and their interaction terms are depicted in Additional file 2: Table S1. There was a significant impact of hyperoxia exposure on both the genotypes in either sex, with an increase in MLI and decrease in RAC upon hyperoxia exposure (Fig. 2B, C). In WT mice, MLI was lower and RAC higher in females compared to males. However, this sex-specific difference was lost in *miR-30a*^−/−^ mice. MLI was higher in *miR-30a*^−/−^ females compared to WT females in the hyperoxia group, with no differences between *miR-30a*^−/−^ males and females. For MLI, genotype, treatment, and sex also showed a main effect with all the interaction terms being significant except for 'treatment x genotype'. Genotype, treatment, but not sex, had a main effect on RAC and all the interaction terms were significant except 'genotype x treatment x sex'.

**Fig. 3:**
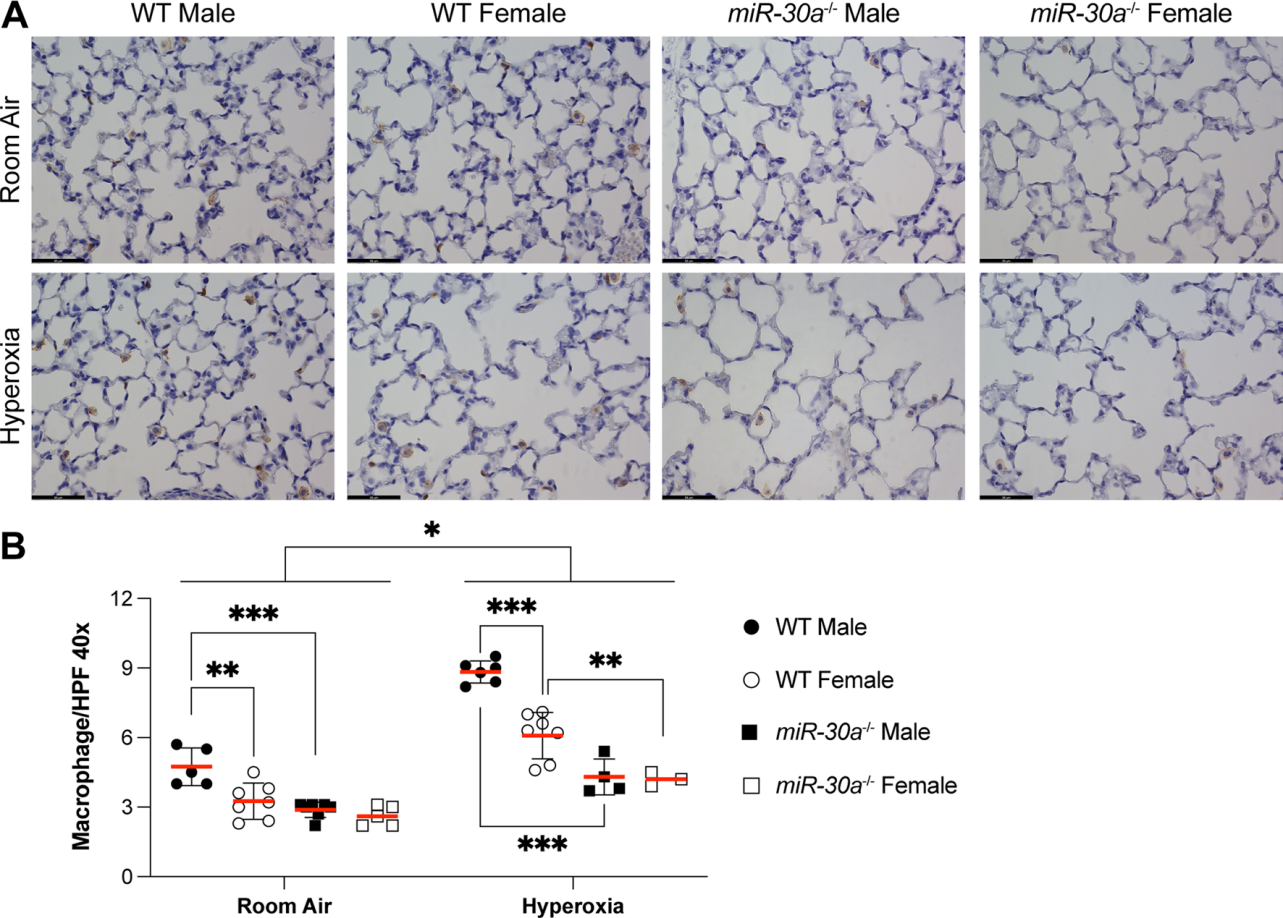
Sex-specific differences in macrophage counts were lost in miR-30a^−/−^ mice in the setting of neonatal hyperoxia exposure. **A** Representative lung sections from male and female WT and *miR-30a*^−/−^ mice in the room air and hyperoxia groups at 40 × magnification stained for macrophages using an F4/80 antibody. Black bars = 50 µm. **B** Macrophage quantitation by immunohistochemistry for F4/80 in neonatal WT and *miR-30a*^−/−^ exposed to room air or hyperoxia (95% FiO_2_, PND1-5) on PND21. Values are mean ± SE. Independent biological replicates in each group are shown (*n = *3–7/group). Significant differences between groups are shown by *P < 0.05, **P < 0.01, and ***P < 0.001. Statistical analysis was performed using 3-way ANOVA

We also quantitated lung macrophages in WT and *miR-30a*^−/−^ mice (Fig. 3A). At baseline, the macrophage count was decreased in WT females (compared to WT males) and in miR*-30a*^−/−^ males (compared to WT males). Hyperoxia increased macrophages in the distal lung in all the hyperoxia exposed groups compared to room air controls. *miR-30a*^−/−^ mice had a lower macrophage count in the distal lung compared to WT mice (Fig. 3B). Main effects were significant for sex, genotype, and treatment by 3-way ANOVA (Additional file 1: Fig. S1). The interaction term of 'sex x genotype' was also significant.

#### Loss of miR-30a leads to similar impairment of distal lung vascular development in male and female mice

**Fig. 4:**
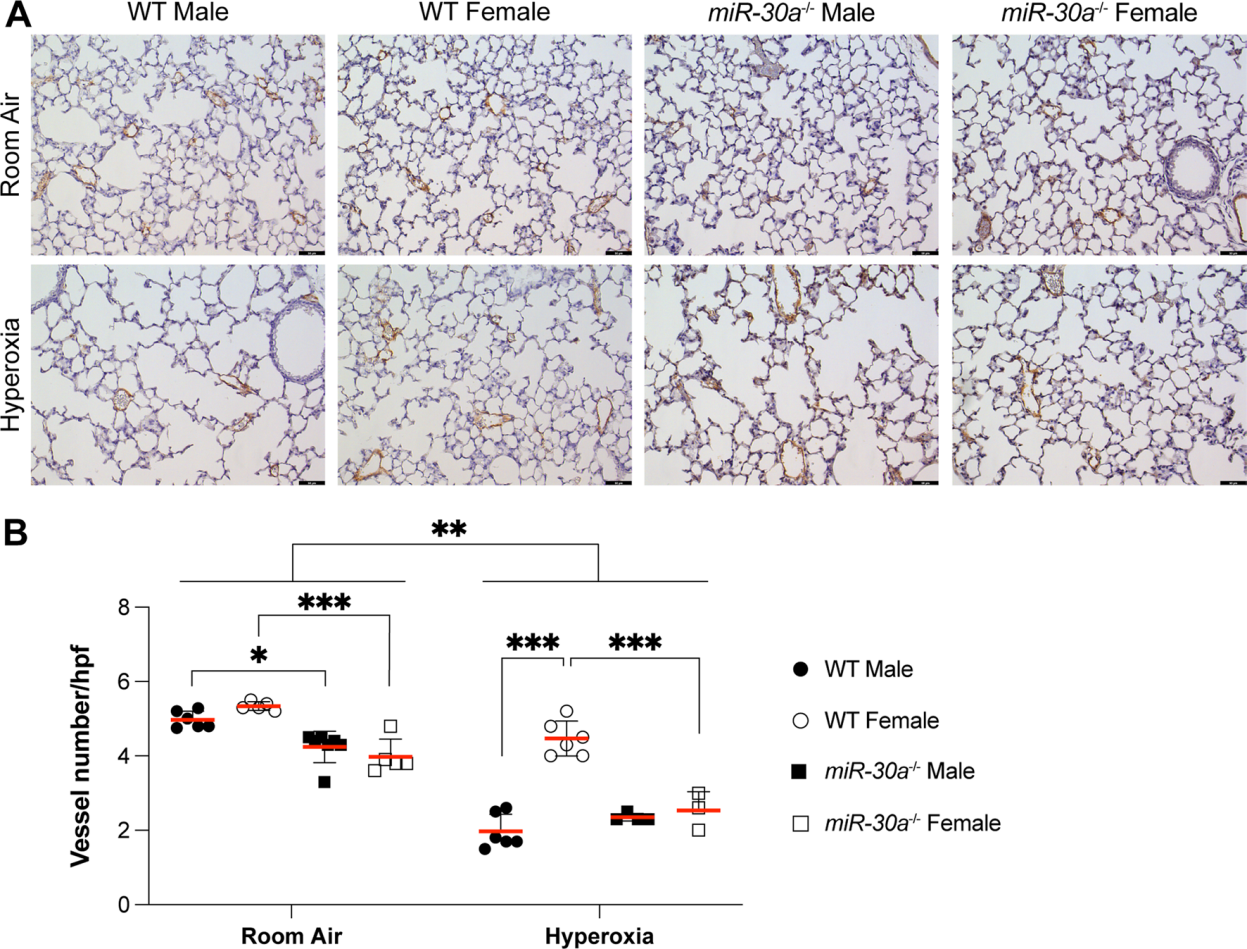
Loss of miR-30a lead to impairment of distal lung vascular development in male and female mice: pulmonary vascular density was assessed in the distal lung using immunohistochemistry for von Willebrand factor (vWF). Brown-stained vessels (< = 50um in diameter) were counted at 20× magnification per high power field (hpf), in 10 randomly chosen fields per lung in neonatal WT and *miR-30a*^−/−^ mice exposed to room air or hyperoxia (95% FiO_2_, PND1-5) on PND 21. **A** Representative immunostained images for von Willebrand factor in male and female WT and *miR-30a*^*−/−*^ mice in the normal air and hyperoxia groups at 20 × magnification. Black bars = 50 µm. **B** Values for counts of pulmonary vessels, are represented as mean ± SE. Independent biological replicates in each group are shown (*n = *3–6/group). Significant differences between groups are shown by *P < 0.05, **P < 0.01, and ***P < 0.001. Statistical analysis was performed using 3-way ANOVA

To assess the effects of hyperoxia on vascular development in the *miR-30a*^−/−^ cohort, vessel counts were quantified using anti-vWF antibodies, as described in methods. Representative lung sections from normoxia- and hyperoxia-exposed WT and *miR-30a*^−/−^ mice are shown in Fig. 4A. At baseline, the vessel count was decreased in *miR-30a*^−/−^ mice compared to WT mice of the same sex. As expected, there was a significant decrease (*P* < 0.01) in vessel count in the hyperoxia group when compared to the room air group (Fig. 4B). Vessel counts in the hyperoxia-exposed *miR-30a*^−/−^ female mice were significantly lower compared to the WT female mice; the same was true for males. Statistical analysis by 3-way ANOVA showed a main effect of treatment and sex, but not genotype (Additional file 1: Fig. S1). All the interaction terms were statistically significant.

### Loss of miR-30a led to significant changes in gene expression in the neonatal lung exposed to hyperoxia

**Fig. 5:**
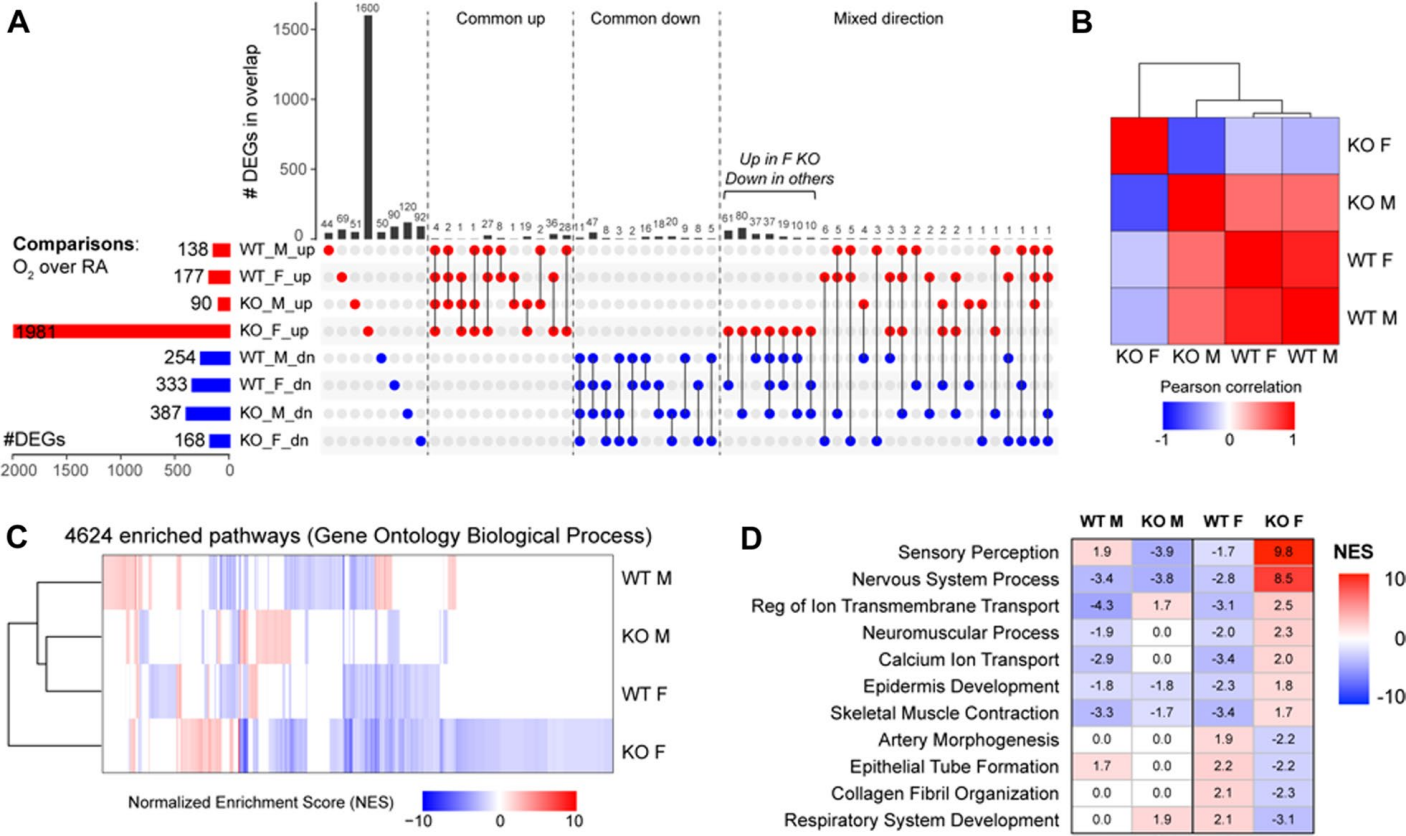
Loss of miR-30a led to significant changes in gene expression in the neonatal lung exposed to hyperoxia. **A** Number of differentially expressed genes in each genotype and sex in the hyperoxia-exposed lungs compared to room air controls. The number of up- and down-regulated genes shared between two or more genotypes, as well as the number of genes modulated in opposite directions between two or more genotypes, are depicted in the upset plot. **B** Correlation patterns of murine lung gene expression signatures scores over a large collection of healthy human lung transcriptomes. For studying the clustering of the lung transcriptome responses based on genotype and sex, we used transcriptome profiles of healthy human adult lung samples (*n = *578) compiled by the GTEx consortium. Summed z-scores for each human individual and each genotype signature were computed and assessed for inter-signature correlations. The *miR-30a*^−/−^ female hyperoxia signature was clearly separated from the other signatures. **C** Pathway-based cluster analysis of gene expression signatures. Gene Set Enrichment Analysis (GSEA) was used to quantify the enrichment of GO Biological Process pathways, and hierarchical clustering and heatmaps were generated using the significant normalized enrichment scores (NES at FDR < 0.25). The *miR-30a*^−/−^ female hyperoxia signature clustered distinctly compared to the other signatures. **D** Biological pathways enriched in WT and *miR-30a*^−/−^ male and female lungs upon exposure to hyperoxia. The highlighted pathways were enriched in the *miR-30a*^−/−^ female but modulated in the opposite direction in the WT female lung

The overlap and differences in gene expression and modulated biological pathways in WT and *miR-30a*^−/−^ mice are highlighted in Fig. 5. The number of differentially expressed genes (DEGs; up- and down-regulated) is shown in Fig. 5A, comparing hyperoxia-exposed animals to controls in each genotype. Strikingly, the *miR-30a*^−/−^ female lung had nearly 2000 up-regulated genes, substantially higher that any of the other groups, leading us to speculate that loss of *miR-30a* has a greater impact on the female neonatal lung compared to the male. Genes that were either unique or common to one or more genotypes are represented in Fig. 5A. Compared to total number of DEGs, there were a minority that overlapped between the genotypes and sexes. Among female mice, there were only 36 up-regulated and 8 down-regulated genes that were common between WT and *miR-30a*^−/−^ mice. To quantitatively derive potential functional relationships between gene signatures, analysis of gene signature summed z-scores in full human lung transcriptomes can capture unappreciated co-regulatory relationships between genes. Whereas large collections of human lung transcriptomes from newborns would be ideal for this purpose, they are not readily available; however, the adult human lung transcriptomes compiled by the GTEx consortium are a suitable alternative [39]. To study the clustering of the lung transcriptome responses based on genotype and sex, we used transcriptome profiles of healthy human adult lung samples (*n = *578) compiled by the GTEx consortium, one of the largest scale efforts to assess genotype/transcriptome relationships in phenotypically healthy individuals [37]. Summed z-scores for each human individual and each genotype signature were computed and assessed for inter-signature correlations. There was a clear separation between the *miR-30a*^−/−^ hyperoxia signatures, as shown in Fig. 5B, with the female *miR-30a*^−/−^ hyperoxia signature showing the most separation. Clustering of enriched biological pathways from Gene Ontology Biological Process, as determined by gene set enrichment analysis (GSEA), also revealed separation of the *miR-30a*^*−/−*^ hyperoxia-exposed female neonatal lung compared to the other genotypes and sexes (Fig. 5C). Among the enriched pathways most induced in *miR-30a*^−/−^ hyperoxia-exposed female neonatal lungs were those related to cell cycle and neuroactive ligand receptor interaction. Lipoprotein assembly and remodeling, PPAR signaling pathway, and the renin angiotensin system were enriched in the *miR-30a*^−/−^ male lung (Additional file 3: Table S2). Since *miR-30a* would be expected to decrease the expression of target genes in the WT female hyperoxia-exposed lung, we identified biological pathways that were negatively enriched in the WT hyperoxia-exposed female lung, but positively enriched in the *miR-30a*^−/−^ female lung. (Fig. 5D). Sensory perception, nervous system process, calcium ion transport, skeletal muscle contraction, and epidermis development were positively enriched in the *miR-30a*^−/−^ female but negatively enriched in the WT female. Conversely, respiratory system development, collagen fibril organization, epithelial tube formation, and artery morphogenesis were negatively enriched in the *miR-30a*^−/−^ female lung but positively enriched in the WT female lung.

**Fig. 6:**
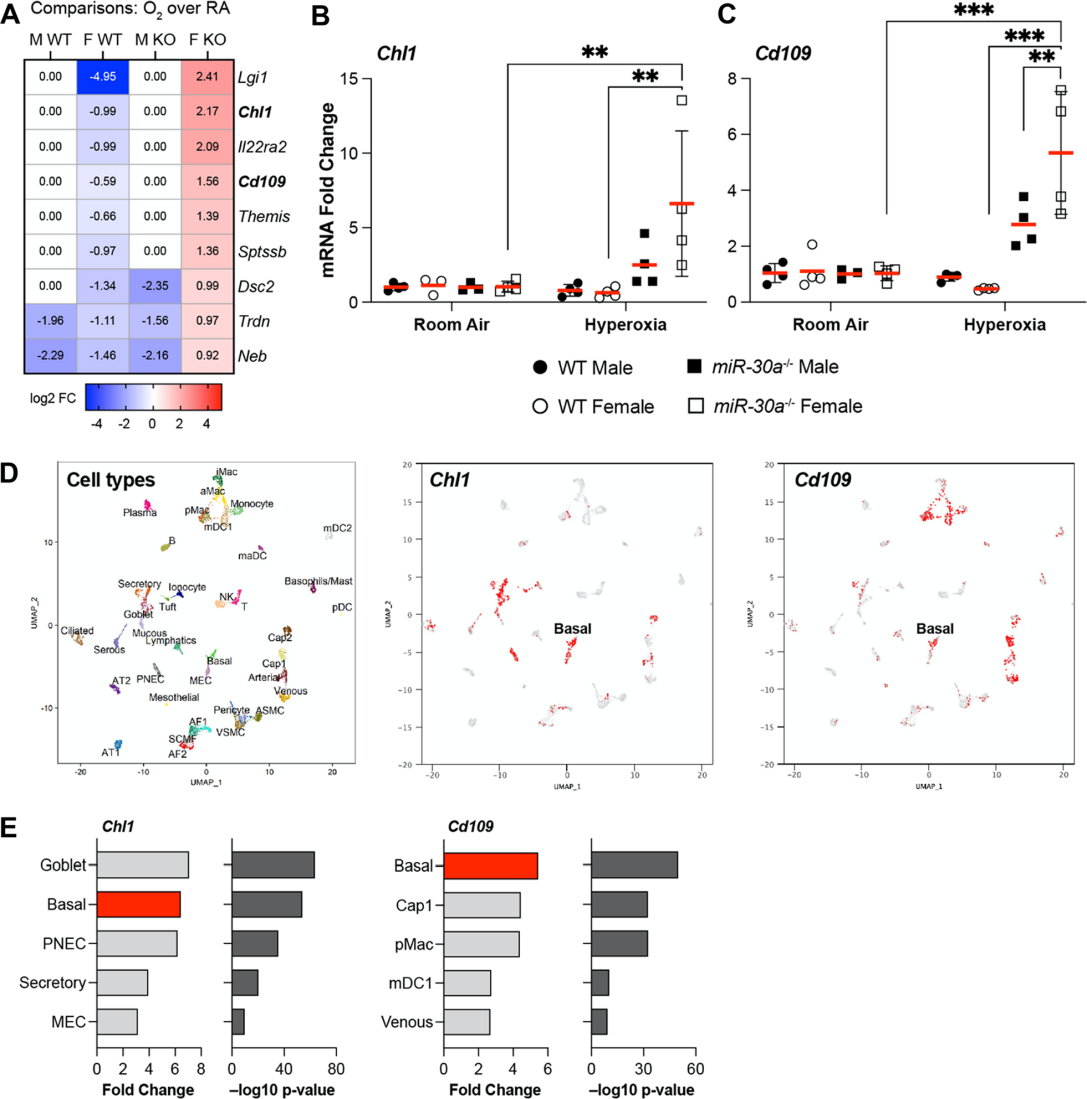
Validation of RNA-Seq data by qRT-PCR.**A** Examples of differentially expressed genes that were upregulated in *miR-30a*^−/−^ female but downregulated in the WT female by RNA-Seq. We selected target genes that were known *miR-30a* targets and down-regulated in the female WT lung but upregulated in the *miR-30a*^−/−^ lung for validation by qRT-PCR. **B** *Chl1* mRNA expression **C** *Cd109* mRNA expression. Values are mean ± SD. Independent biological replicates in each group are shown (*n = *3–4/group). Statistical analysis was performed using 3-way ANOVA. Significant differences between groups are shown by **P < 0.01, and ***P < 0.001. **D** Cell-type specific expression of *Chl1* and *Cd109* from the LungMAP database. UMAP plots showing different lung cell sub-populations and expression of *Chl1* and *Cd109* in basal cells. **E** Expression levels of *Chl1* and *Cd109* (Fold change and -log _10_ p value) in the lung cell types. The cell groups with the highest expression are depicted here. PNEC: pulmonary neuroendocrine cell; MEC: myoepithelial cell; Cap1: general capillary endothelial cell; pMAC: tissue resident macrophage; mDC1: classical dendritic cell subset 1

Figure 6A highlights examples of differentially expressed genes that were upregulated in *miR-30a*^−/−^ females but downregulated in the WT female upon exposure to hyperoxia compared to controls maintained in room air. We selected genes that were known *miR-30a* targets and down-regulated in the female WT lung but upregulated in the female *miR-30a*^−/−^ lung for validation by qRT-PCR in an independent cohort of mice, similarly, exposed to hyperoxia and euthanized on PND21. One of the targets we validated was *Chl1* (Cell Adhesion Molecule L1 Like), a protein that functions as an extracellular matrix and cell adhesion protein and potentiates integrin-dependent cell migration towards extracellular matrix proteins. *Chl1* expression was increased in *miR-30a*^−/−^ hyperoxia-exposed females compared to its room air control and compared to WT hyperoxia-exposed females (Fig. 6B). In the LungMAP database, the *Chl1 gene* was expressed in airway basal cells, goblet cells, and PNECs (Fig. 6D, E).

Another gene target that we validated was *Cd109*, which encodes a glycosyl phosphatidylinositol (GPI)-linked glycoprotein on the surface of platelets, activated T-cells, and endothelial cells. The protein negatively regulates signaling by transforming growth factor beta (TGF-beta) by binding to it. In our validation by qRT-PCR, *Cd109* expression was increased in *miR-30a*^−/−^ hyperoxia-exposed females compared to its room air control, compared to WT hyperoxia-exposed females, and compared to *miR-30a*^−/−^ hyperoxia-exposed males (Fig. 6C). In the LungMAP database, the *Cd109* gene was highly expressed in the basal cells in the human lung airway in addition to other cell types (Fig. 6D, E).

### Gene expression signature in the miR-30a^−/−^ female lung associated with human BPD blood transcriptomes

**Fig. 7:**
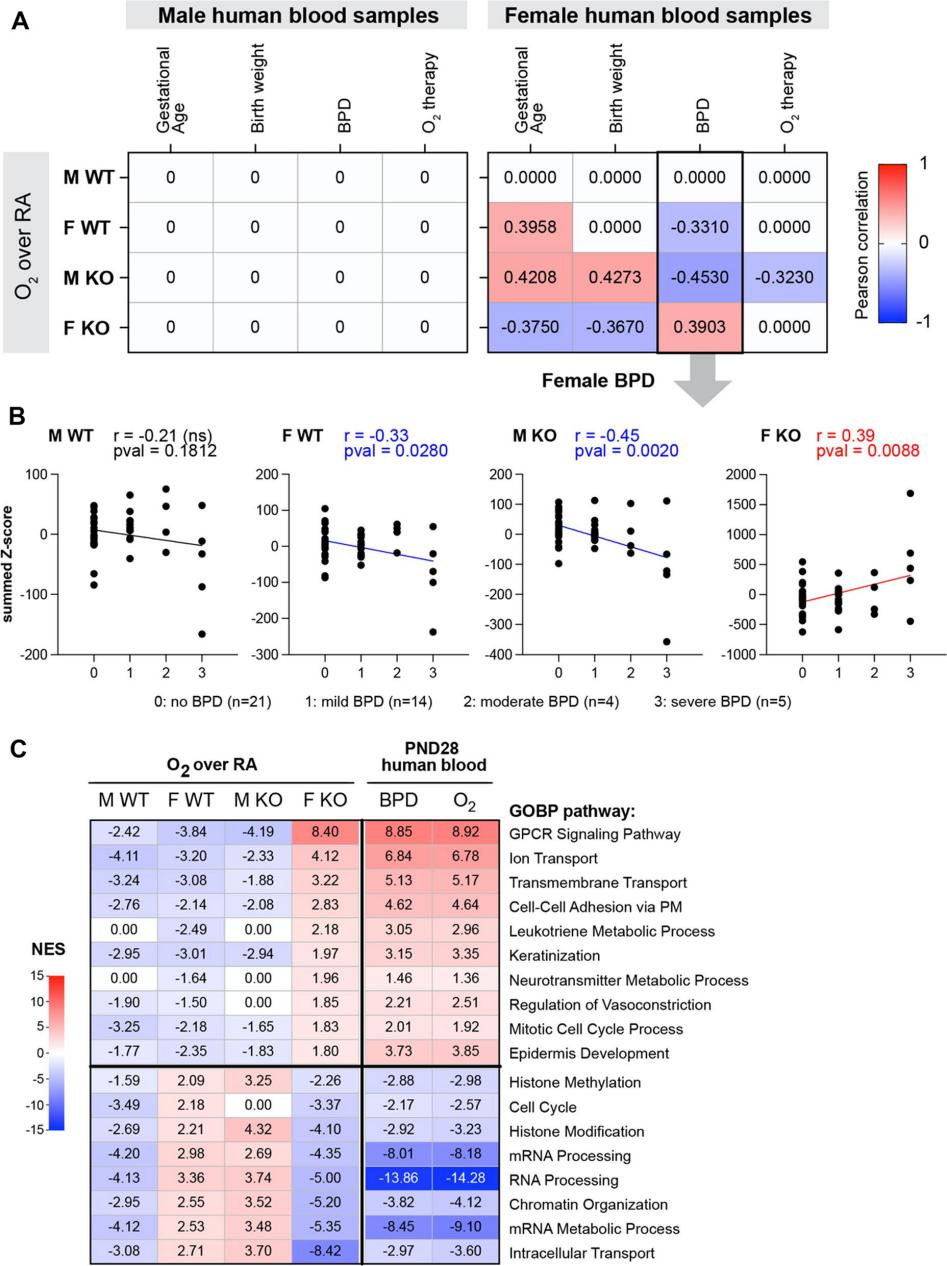
Gene expression signature in the miR-30a^−/−^ female lung associated with human BPD blood transcriptomes. **A** The distributions of summed z-scores for the hyperoxia gene signatures from the four genotypes at PND21 were compared to the gene expression signatures obtained from the blood of human preterm neonates who developed BPD. In the human study, 111 preterm neonates of < 32 weeks gestation were included in the study. 43 neonates had no BPD, 40 had mild BPD, 13 had moderate and 15 had severe BPD. Disease severity was graded according to criteria proposed by Jobe and Bancalari [80]. Pearson correlation coefficient values are shown, correlating hyperoxia signatures to each clinical variable (gestational age, birth weight, BPD status, and oxygen requirement). **B** Distribution of summed z-scores (y-axis) for the murine lung hyperoxia signatures were correlated with the human newborn blood samples collected at PND28, stratified by biological sex and by BPD status (x-axis: BPD severity; no BPD, mild, moderate, and severe). Association was evaluated using the parametric Pearson correlation, with the Pearson correlation coefficient (r) and *p*-values indicated. **C** Selected Gene Ontology pathways and their normalized enrichment scores observed in the blood transcriptome of BPD patients at PND28, and the murine lung exposed to hyperoxia (WT and *miR-30a*^−/−^). Biological pathways that were correlated between the *miR-30a*^−/−^ female lung signature with the human BPD patients, while modulated in the opposite direction in WT female lung are highlighted

The distribution of summed z-scores for the hyperoxia gene signatures from the four genotypes at PND21 was compared to gene expression signatures obtained from the blood of human preterm neonates who developed BPD [38]. Four clinical variables were chosen: gestational age, birth weight, BPD status, and oxygen requirement at 28 days and the samples were stratified by sex (Fig. 7A). The WT female hyperoxia signature positively correlated with gestational age, and negatively with BPD status. Interestingly, the gene signature pattern in *miR-30a*^−/−^ males correlated positively with both gestational age and birth weight and negatively with BPD status and oxygen therapy at 28 days. Strikingly, the transcriptomic signature from the hyperoxia-exposed *miR-30a*^−/−^ female lungs had a negative correlation with gestational age and birth weight and a positive correlation with BPD status. The correlation values based on BPD disease severity are shown in Fig. 7B. Distribution of summed z-scores (y-axis) for the murine lung hyperoxia signatures were correlated with the gene signature in human newborn blood samples collected at PND28, stratified by biological sex and by BPD status (x-axis: BPD severity; no BPD, mild, moderate, and severe). A significant negative correlation with either the WT female or *miR-30a*^−/−^ male hyperoxia signatures, but a significant positive correlation with the *miR-30a*^−/−^ female hyperoxia signature. We next identified biological pathways that were similarly enriched between the *miR-30a*^−/−^ female lung hyperoxia exposure and the human BPD patients (compared to healthy controls) but modulated in the opposite direction by hyperoxia in the WT female lung (Fig. 7C). Pathways related to keratinization, ion transport, neurotransmitter metabolic process, and regulation of vasoconstriction were positively enriched in the *miR30a*^−/−^ female lung and human BPD, while histone methylation and chromatin organization were negatively enriched in both.

### miR-30a was expressed in the bronchioles in the lung upon exposure to hyperoxia compared to vessels and the lung parenchyma

**Fig. 8:**
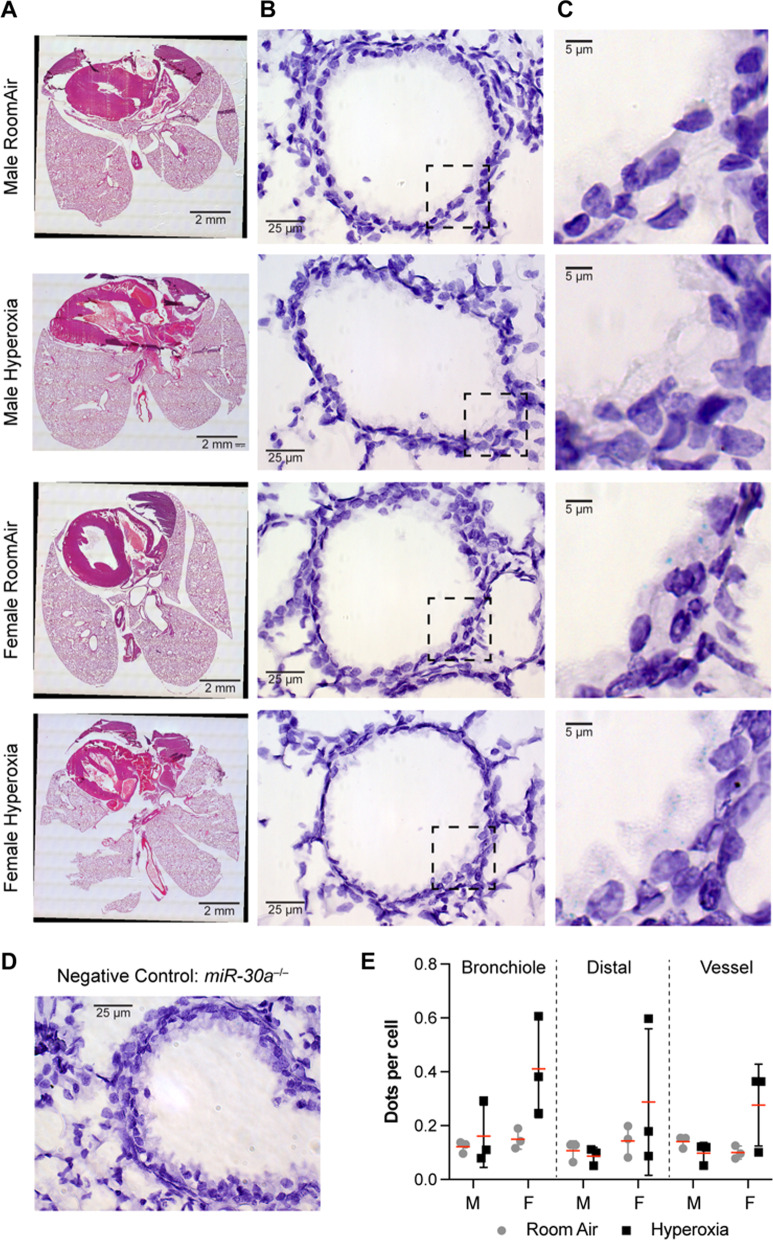
miR-30a was expressed in the bronchiolar epithelial cell population in the lung upon exposure to hyperoxia: In situ, hybridization in representative WT lung sections for miR-*30a* identified the spatial localization of *miR-30a* in the lung in hematoxylin-stained sections, with consecutive sections stained with eosin and hematoxylin for lung structure. **A** Tiled H&E image of the entire lung section subjected to *miR-30a *in situ hybridization. Black bars = 2 mm. **B** Representative lung regions (40×) focused on bronchioles to discern *miR-30a* expression pattern in the lung. Black bars = 25 µm. **C** Magnified (60×) images of the bronchioles showing the punctate light blue staining of *miR-30a* expression in the bronchial epithelial cells, highest in the WT female lung exposed to hyperoxia. Black bars = 5 µm. **D** Representative lung section from a *miR-30a*^−/−^ mouse showing absence of punctate staining. Black bar = 25 µm. **E** Quantitation of *miR-30a* staining in the lung in the different anatomical regions (bronchioles, lung parenchyma and vessel) for males (M) or females (F) exposed to room air (grey circles) or hyperoxia (black squares). *N* = 3/group

To delineate cell-specific expression of *miR-30a* in the murine lung we performed in situ hybridization in lung sections, shown in Fig. 8. The expression was mainly localized to the bronchiolar epithelial cells (Fig. 8B, C, E). Expression was also measured in the vessels and the lung parenchyma, but the expression was lower compared to the bronchiolar epithelium. Overall, the expression was higher in the female lung after exposure to hyperoxia in the bronchioles but was not statistically significant.

## Discussion

Neonatal male mice show greater arrest in alveolarization and vascularization following exposure to hyperoxia when compared to females [18]. This protective effect in female mice is accompanied by increased *miR-30a* expression [26]. In this investigation, we examined the role of *miR-30a* in alveolarization and vascular development and showed that *miR-30a* modulates sex-specific differences in neonatal lung in hyperoxic conditions. Significantly, *miR-30a*^−/−^ mice did not exhibit sex-specific differences in alveolarization and vascular development in the postnatal hyperoxia model, as previously reported in WT mice [18]. Without expression of *miR-30a,* both sexes suffered similar adverse impact in alveolarization and vascular development when exposed to hyperoxia.

Post-transcriptional regulation of many protein-coding genes is modulated by small noncoding RNAs, called miRNAs (miRs). They mainly act to decrease gene expression of their target genes by decreasing mRNA stability. Each miRNA can have up to hundreds of downstream target genes and individual genes can be modulated by multiple miRs. Differential expression of miRNAs could be a potential mechanism underlying sexual dimorphism in a variety of diseases. Bronchopulmonary dysplasia has a multifactorial etiology and is a disease that specifically afflicts the developing preterm lung. Males are more susceptible to the development of this disease, which suggests that sex as a biological variable plays a crucial role in its pathophysiology [40]. Many studies have emphasized the role of miRs in the pathophysiology of BPD, however they did not address the sex-specific differences in their expression or regulation [41–50]. *miR-30a* emerged as one of the possible factors mediating the sex-specific differences in our previous study [26]. In vivo*,* the hyperoxia-exposed female lung showed higher *miR-30a* expression and in vitro female human pulmonary microvascular endothelial cells showed increased expression of *miR-30a* upon exposure to hyperoxia. *miR-30a* has anti-inflammatory [51, 52] and anti-fibrotic [53, 54] effects in various organ systems and disease processes.

Multiple publications have investigated the role of *miR-30a* in pulmonary diseases. *miR-30a* was down-regulated in the bronchoalveolar lavage fluid and lung samples obtained from patients with idiopathic pulmonary fibrosis (IPF) [55–57]. The adverse phenotype observed in the *miR-30a*^−/−^ lung could be attributed to many downstream targets with relevance to lung diseases including idiopathic pulmonary fibrosis, COPD, and asthma [58, 59]. *miR-30a* stabilizes pulmonary vessels by decreasing vascular hyperpermeability [60] and was downregulated in a model of hypoxia-induced pulmonary hypertension [61]. In a case control study, which reported the expression profiles of miRNAs in the peripheral blood of very low birth weight preterm infants, *miR-30a* expression was decreased both in babies with evolving BPD at 2 weeks postnatal age and at 36 weeks of post-menstrual age. [21].

We noted the macrophage count in the hyperoxia exposed *miR-30a*^−/−^ lungs to be lower than hyperoxia exposed WT mice. The macrophage population within the lung is very heterogenous with unique niches and biological roles [62–64]. *miR-30a* plays a role in macrophage polarization [65–67] and the distinction between alveolar and interstitial macrophages in the *miR-30a*^−/−^ lung and macrophage polarization state needs to be investigated in future experiments. Interestingly, pathways related to sensory perception and nervous system process were enriched in the *miR-30a*^−/−^ female lung upon exposure to hyperoxia compared to the WT female lung. Among the genes upregulated in the *miR-30a*^−/−^ hyperoxia- exposed female lung are those enriched in pulmonary neuroendocrine cells (PNECs). PNECs have important roles in airway physiology and immunity by producing bioactive amines and neuropeptides [68]. Many pulmonary diseases exhibit PNEC hyperplasia [69, 70]. These include genes such as chromogranin A (*Chga*), chromogranin B (*Chgb*), secretogranin-II (*Scg2*), Ubiquitin C-Terminal Hydrolase L1 (*Uchl1*), olfactory receptor genes (*Olfr 78*), calcitonin related genes (*Calcb* and *Calcr*) and Neural cell adhesion molecule 1 (*Ncam 1*) [70–73]. *Tubb3* expression was also higher. Airway basal stem cells give rise to *Tubb 3* + PNECs and *Tubb 3* + PNECs are increased in infant diseases [73]. A study in infants with BPD also revealed a higher number of pulmonary neuroendocrine cells [74]. Basal cell markers, such as *Krt5* and *Trp63,* were also upregulated in the *miR-30a*^−/−^ hyperoxia-exposed female lung [65]. Basal cells function as tissue-specific stem cells participating in repair after lung injury. The biological relevance of these genes in the *miR-30a*^−/−^ hyperoxia-exposed female mice needs to be examined in future studies. Interestingly, the neurotransmitter metabolic process was a biologic pathway enriched in the blood transcriptomic signature of preterm babies with BPD and the *miR-30a*^−/−^ hyperoxia- exposed female lung.

We validated two genes (*Cd109 and Chl1*) that were predicted to be *miR-30a* targets and were upregulated in the *miR-30a*^−/−^ and downregulated in the WT hyperoxia-exposed neonatal female lung. In a model of oxygen-induced retinopathy, Xu et al*.* reported that CD109 was among the most down-regulated proteins in hypoxic retinal endothelial cells. Overexpression of CD109 decreased, while downregulation increased endothelial cell proliferation in both human retinal and umbilical vein endothelial cells [75]. CD109 is a GPI-anchored protein that acts as a TGF-β co-receptor and a negative regulator of TGF-β signaling. It facilitates TGF-beta receptor endocytosis and subsequent degradation, thus inhibiting TGF-beta signaling [76]. *Chl1* is expressed in the mouse carotid body, and loss of *Chl1* decreased mortality and improved adaptation to acute hypoxia [77]. In a study involving pulmonary adaption to hypoxia at high altitude, *Chl1* was one of the most downregulated genes, possibly modulating the enhanced ventilatory response after exposure to hypoxia. [78] These target genes are expressed in the airway epithelium, basal cells, and PNECs as shown from the LungMAP database. Furthermore, ISH studies from this study localized *miR-30a* in the bronchioles. *miR-30a* expression was increased in bronchial brushings, and *miR-30a* transcripts were localized to human bronchial epithelium [79]. PNECs are found at airway bifurcations or bronchioloalveolar junctions and serve as airway sensors to control lung immune response [68, 73]. Taken together, these data suggest a possible role of *miR-30a* in the pulmonary basal cells and/or PNECs.

We recognize the limitations of this current investigation. The findings in this study are from a global knock-out/loss of *miR-30a*. Intrapulmonary delivery strategies for inhibiting *miR-30a* or cell-specific loss of *mir-30a* would be superior to elucidate the organ-specific or cell-specific role of *miR-30a*. Long-term functional consequences of *miR-30a* loss related to lung function and pulmonary hypertension were not measured in this study. The cell-specific expression data in human lungs may be tightly associated with the developmental stage of the human lung and needs further validation. The correlation between mouse lung samples and human blood samples is not ideal, but these findings lend translational relevance of the biological role of *miR-30a* in BPD pathogenesis. Additionally, findings from the murine model may not translate directly to humans. However, several parallels between *miR-30a* expression in human patients with other lung diseases (COPD, asthma, and IPF) that possess similarities in phenotypes to BPD support the translational relevance of this miRNA [57, 59, 65]. In conclusion, we show the loss of sex-specific differences in neonatal hyperoxic lung injury after loss of *miR-30a* and a significant impact of *miR-30a* loss in the female neonatal lung. We also highlight the enrichment of genes expressed in PNECs and airway basal cells in *miR-30a-/-* female lungs exposed to hyperoxia.

## Perspectives and significance

We believe our data present a significant advancement in explaining the molecular basis behind the sex-specific differences of neonatal hyperoxic lung injury and BPD. The primary mechanisms of *miR-30a* regulation and functional outcomes remain elusive. Exploring whether supplementation of *miR-30a* through AAV-mediated or other approaches can rescue or improve the lung injury phenotype in the murine hyperoxic lung injury model should be the focus of future research.

## Conclusions

*miR-30a* could be one of the biological factors mediating resilience of the preterm lung to neonatal hyperoxic lung injury. A better understanding of the effects of *miR-30a* on pulmonary angiogenesis and alveolarization may lead to novel therapeutics for the treatment of BPD.

### Supplementary Information

**Additional file 1.** 3-way ANOVA analysis.

**Additional file 2: Table S1.** Differentially expressed genes (Hyperoxia over Room Air).

**Additional file 3: Table S2.** Gene Set Enrichment Analysis (GSEA).

## Data Availability

The *miR-30a*^*−/−*^ data have been submitted to NCBI GEO (GSE213287). Previous WT samples were submitted to NCBI GEO (GSE97804). The datasets used and/or analyzed during the current study are available from the corresponding author on reasonable request**.** Bronchopulmonary dysplasia (BPD) blood transcriptomics from newborns were downloaded from the public NCBI GEO dataset GSE32472.
