# Supplementary figures and images for: Loss of microRNA-30a and sex-specific effects on the neonatal hyperoxic lung injury
Source: Biol Sex Differ. 2023 Aug 8;14:50. doi: 10.1186/s13293-023-00535-6 (PMC10408139; doi:10.1186/s13293-023-00535-6)

Figure S1

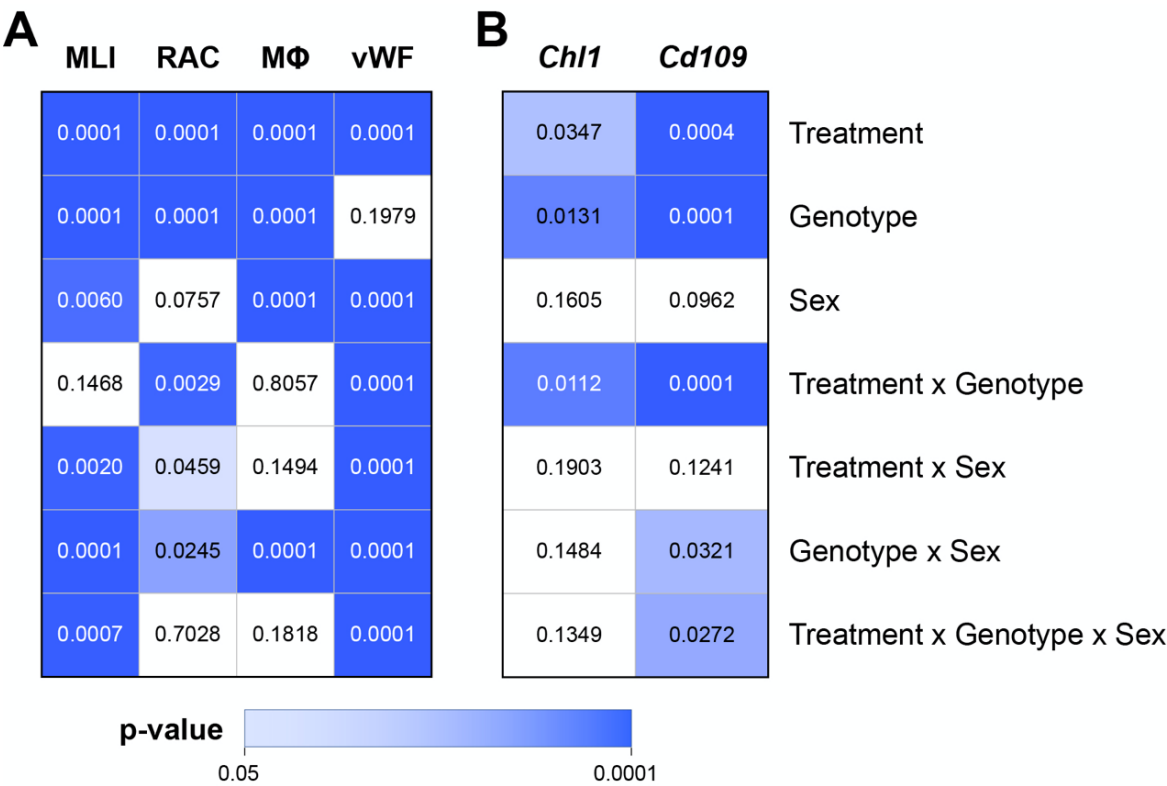

Supplement: Supplementary file 1 — Additional file 1. 3-way ANOVA analysis. [file 13293_2023_535_MOESM1_ESM.pdf]
